# Novel mutation in *ENG* gene causing Hereditary Hemorrhagic Telangiectasia in a Peruvian family

**DOI:** 10.1590/1678-4685-GMB-2019-0126

**Published:** 2020-02-27

**Authors:** Alejandro Zevallos-Morales, Alexis Murillo, Milagros M. Dueñas-Roque, Ana Prötzel, Luis Venegas-Tresierra, Verónica Ángeles-Villalba, Miguel Guevara-Cruz, Ada Chávez-Gil, Ricardo Fujita, Maria L. Guevara-Fujita

**Affiliations:** 1 Universidad de San Martín de Porres, Facultad de Medicina Humana, Centro de Genética y Biología Molecular, Lima, Peru.; 2 Hospital Nacional Edgardo Rebagliati Martins, EsSalud, Lima, Peru.

**Keywords:** ENG, Hereditary Hemorrhagic Telangiectasia, Osler-Weber-Rendu disease

## Abstract

Hereditary Hemorrhagic Telangiectasia (HHT) is a rare disorder of vascular development. Common manifestations include epistaxis, telangiectasias and arteriovenous malformations (AVMs) in multiple organs. Most patients have deletions or missense mutations in the *ENG* or *ACVRL1* gene respectively, significantly affecting endothelium homeostasis. We analyzed the *ENG* gene in five members of a Peruvian family affected by HHT. One novel mutation was found in exon four of the *ENG* gene c.408delA, at aminoacid residue 136. This mutation changes the subsequent reading frame producing an early stop at residue 162, preserving only one fourth of the normal protein of 658 aa. This mutation was found in the four affected members of family.

Hereditary Hemorrhagic Telangiectasia (HHT), also known as Osler-Weber-Rendu disease (OMIM #187300) is an autosomal dominant and multisystemic vascular dysplasia characterized by telangiectasias and arteriovenous malformations (AVMs) in major organs (Miller *et al.*, 1988; Begbie *et al.*, 2003; Abdalla and Letarte, 2006; Richards-Yutz *et al.*, 2010; Faughnan *et al.*, 2011; McDonald *et al.*, 2011). These AVMs commonly appear in lungs, brain and liver, and are pathological connections between arteries and veins, lacking the capillary bed (Begbie *et al.*, 2003).

Patients with HHT can suffer from a plethora of symptoms. Usually, they manifest with recurrent epistaxis and telangiectasias but can also present more severe symptoms as anemia, gastrointestinal bleeding and complications due to AVMs in the brain, liver or lungs (McDonald *et al.*, 2011). The diagnosis of HHT is made using the Curaçao criteria that consist of epistaxis, telangiectasias, visceral lesions and family history; to diagnose it, three of the four Curaçao criteria should be present (Faughnan *et al.*, 2011).

Several subtypes of HHT have been described, each one related to a different gene/locus. The most frequent subtypes, HHT1 and HHT2, are caused by mutations in the *ENG* and *ACVRL1* genes respectively which account for almost 90% of all HHT cases (Richards-Yutz *et al.*, 2010). HHT3 and HHT4 have been mapped to chromosomal regions 5q31.3-q32 and 7p14 respectively, but no gene or specific clinical manifestations have been identified yet (Cole *et al.*, 2005; Bayrak-Toydemir *et al.*, 2006). Mutations in *GDF2* gene have been related to a HHT-like disease (Wooderchak-Donahue *et al.*, 2013; McDonald *et al.*, 2015). Finally, another gene called *SMAD4*, has also been identified as responsible for a type of HHT which presents with juvenile polyposis (Richards-Yutz *et al.*, 2010; McDonald *et al.*, 2011) .

We collected blood samples from five individuals**;** four affected with HHT (IV-2, V-1, V-2, VI-1) and one unaffected (VI-2), belonging to a native Peruvian family of 20 individuals reported across 6 generations bearing HHT (Figure 1). Patient IV-2, our proband, was diagnosed with HHT at 48 years of age. She has history of a brain abscess at 33 years old, multiple telangiectasias in her tongue, hand, stomach and esophagus, and a gastric ulcer. Her daughter (V-2) was diagnosed with HHT at 18 years of age. She also has a history of a brain abscess at 28 years of age. The other affected members of the family (V-1 and VI-1) were suspected for HHT because of recurrent epistaxis. Patient V-1 died of sepsis, due to intraabdominal infection, months after the blood sample was extracted. The other affected members of the family (I-1, II-2, III-4, III-5, and IV-3) had already died at the time of the interview, and were reported with recurrent epistaxis by reference of relatives.

**Figure 1:**
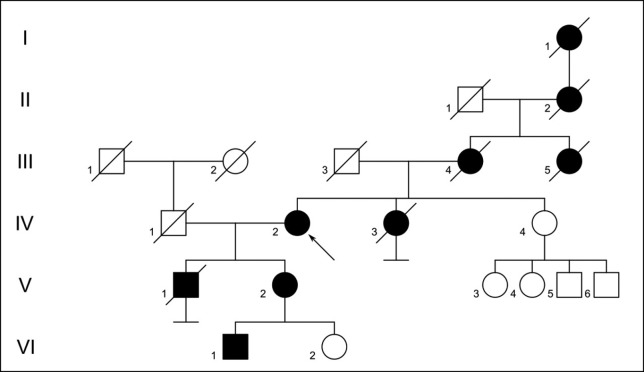
Pedigree of the affected family. In black, individuals with reported HHT, in open, individuals with no HHT symptoms. The black arrow indicates the proband.

We chose to analyze the *ENG* gene first because of the clinical characteristics of HHT1 presented in some affected individuals. (Table 1) Patients IV-2 and V-2 had been hospitalized due to brain abscess, a rare manifestation commonly associated with HHT1 (Girod *et al.*, 2007; Sell *et al.*, 2008). The manifestations of respiratory problems, such as dyspnea and digital clubbing typical for HHT1, were also present in those individuals (Lesca *et al.*, 2007; Sabba *et al.*, 2007; Mu *et al.*, 2018).

**Table 1:** Clinical findings in the four living affected individuals

Clinical Characteristics	IV-2	V-1^*^	V-2	VI-1
**Head & Neck**				
*Nose*				
Spontaneous, recurrent epistaxis (onset childhood)	Yes	Yes	Yes	Yes
Nasal mucosa telangiectases	Yes	Yes	Yes	Yes
*Mouth*				
Lip telangiectases				
Tongue telangiectases	Yes		Yes	
Palate telangiectases			Yes	
**Cardiovascular**				
*Vascular*				
Venous varicosities	Yes		Yes	
**Respiratory**				
Dyspnea	Yes	Yes	Yes	
Cyanosis	Yes		Yes	
**Abdomen**				
*Gastrointestinal*				
GI hemorrhage (onset usually in 5th -6th decade)	Yes			
Angiodysplasia	Yes			
Telangiectases (stomach, duodenum small bowel and colon)	Yes			
Melena	Yes			
Hematochezia	Yes			
Hematemesis	Yes			
**Skeletal**				
*Hands*				
Finger pad telangiectases			Yes	
Clubbing	Yes		Yes	
**Skin**, **Nails**, **& Hair**				
*Skin*				
Telangiectases (specially on tongue, lips, palate, fingers, conjunctiva, trunk, nail beds, and fingerprints)	Yes		Yes	
**Neurologic**				
Migraine headache	Yes		Yes	Yes
Seizures	Yes			
Brain Abscess	Yes		Yes	
**Hematology**				
Anemia	Yes			
Polycythemia			Yes	

* Patient died after clinical history was retrieved and blood sample given.

To discard *a priori* the involvement of other genes out of *ENG*/HHT1, we checked that none of the patients had any liver involvement, a characteristic frequently found in patients with HHT2 (Letteboer *et al.*, 2006; Lesca *et al.*, 2007). No intestinal polyps (specific of *SMAD4),* were found in any patient. Finally*,* mutations in *GDF2* are rare and no correlations to specific clinical findings have been reported (Wooderchak-Donahue *et al.*, 2013). Gastrointestinal symptoms were present in patient IV-2, but not in the other affected family members.

We designed primers for the 15 exons of *ENG* (NM_000118.3 GRCh37). The primers were manufactured by LGC Biosearch Technologies (Petaluma, CA). Genomic DNA was extracted from peripheral blood using the salting-out procedure with some modifications. (Miller *et al.*, 1988). Specific conditions were designed for Polymerase Chain Reaction (PCR) amplification. PCR products were purified using GeneJET PCR Purification Kit (Thermo Fisher Scientific, Boston, MA, USA). Sanger sequencing was performed with purified PCR products using BigDye Terminator v3.1 Cycle Sequencing Kit and the ABIPRISM 3500 Genetic Analyzer (Applied Biosystems, Foster City, CA, USA). The generated sequences were analyzed using FinchTV software compared to the GRCh37.p13 assembly.

We found two mutations in the *ENG* gene, both of them were heterozygous. A single nucleotide deletion in exon 4 (c.408delA) in the affected members of the family (IV-2, V-1, V-2, VI-1). The mutation was not previously reported and was missing in the unaffected individual (VI-2). We also found an insertion c.991+26_991+27insCCTCCC of 6bp at intron 7. The insertion was only found in one patient IV-2 and has been reported previously (rs148063362) as a benign variation without clinical significance.

The novel 1 bp deletion we report causes a frameshift in exon four at aminoacid residue 136, generating an early termination of the protein at aminoacid position 162 instead of the normal length protein of 658 aa. This truncated protein lacks the main functional endoglin sites residues 270-282 that are essential for junction with GDF2, a major component in the TGF-ß signaling pathway; and the cell attachment site at residues 399-401. (Figure 2) The mutation was submitted to ClinVar and accepted in January 2018 with the identification SCV000681418.1.

**Figure 2:**
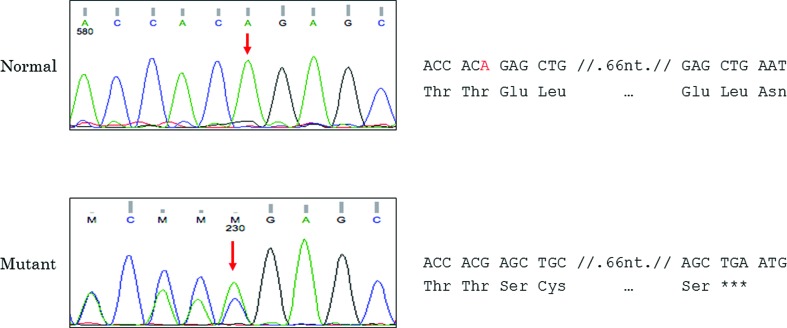
Normal and mutated exon 4 sequences of the *ENG* gene. These sequences were obtained with a reverse primer. The right panel shows both sequences translated, showing an early termination in the mutated case.

Several cases of brain abscess in HHT patients have been described, (Sabba *et al.*, 2007; Sel *et al.*, 2008) but very few of them with genetic analysis. We found a case report in which the patient only manifested with a brain abscess and after a more detailed clinical history, it was found to have recurrent epistaxis as other family members. Genetic analysis showed a 13-nucleotide duplication in exon 6 of *ENG*, causing a frameshift and further protein truncation*.* (Chen and Lin, 2013)

Mutations found in *ENG* are heterozygous, a review of mutations causing HHT1 identified that most mutations were frameshift deletions (32.9%), causing an early stop codon and truncating the protein (Abdalla and Letarte, 2006). Initially, it was believed that the truncated endoglin would interfere in the normal function of the protein by a gain-of-function process. However, it has been proved that in most cases the mutated endoglin is not expressed in the surface of the cells and in some situations, a small amount of mutated protein can be measured in the intracellular space. (Pece *et al.*, 1997; Pece-Barbara *et al.*, 1999) Further, no homozygous mutations have been reported in patients with HHT1, but studies with animals have shown that are incompatible with life. (Abdalla and Letarte, 2006) Because of this, the current model of disease pathogenesis for HHT has been associated with haploinsufficiency, meaning that the quantity of the remaining protein is incapable of covering for the cellular needs (Gordon and Blobe, 2008). The lack of the normal amount of endoglin would hinder its activation through the BMP9/GDF2 complex, subsequently disrupting the activation of different SMAD pathways which are part of the TGF-ß signaling pathway (Begbie *et al.*, 2003; Abdalla and Letarte, 2006; Shovlin, 2010; McDonald *et al.*, 2015). This cascade and signaling pathway, regulates several cellular processes in vascular endothelium such as proliferation, differentiation, migration, and survival (Gordon and Blobe, 2008).

Nonetheless, haploinsufficiency, cannot explain the focal distribution of the disease by itself. (Ruiz-Llorente *et al.*, 2017) In HHT, there is a limited number of AVMs affecting particular organs rather than a systemic manifestation. This is why, Kudson’s two-hit mechanism, has been proposed as an alternative. This means that a patient with a germline mutation, in any of the HHT related genes, would need a trigger such as ischemia, inflammation, vascular lesion or somatic mutation to develop the disease. (Ruiz-Llorente *et al.*, 2017) Supporting this mechanism, somatic mutations have been found in telangiectasias from HHT patients with germline mutations. Most frequently, somatic mutations and germline mutations, are frameshift mutations. (Snellings *et al.*, 2019)

In conclusion, we found a novel mutation c.408delA (p.Glu137SerfsTer26) in the *ENG* gene causing a codon frameshift and generating an early termination (aminoacid residue 132) of the endoglin protein causing HHT1in four affected members of a Peruvian family.
